# Inactivation of SARS-CoV-2 on surfaces and in solution with Virusend (TX-10), a novel disinfectant

**DOI:** 10.1099/acmi.0.000228

**Published:** 2021-04-26

**Authors:** Enyia R. Anderson, Grant L. Hughes, Edward I. Patterson

**Affiliations:** ^1^​ Departments of Vector Biology and Tropical Disease Biology, Centre for Neglected Tropical Disease, Liverpool School of Tropical Medicine, Liverpool L3 5QA, UK; ^2^​ Department of Biological Sciences, Brock University, St. Catharines L2S 3A1, ON, Canada

**Keywords:** SARS-CoV-2, COVID-19, Virusend, TX-10, inactivation, disinfectant

## Abstract

Until an effective vaccine against SARS-CoV-2 is available on a widespread scale, the control of the COVID-19 pandemic is reliant upon effective pandemic control measures. The ability of SARS-CoV-2 to remain viable on surfaces and in aerosols, means indirect contact transmission can occur and there is an opportunity to reduce transmission using effective disinfectants in public and communal spaces. Virusend (TX-10), a novel disinfectant, has been developed as a highly effective disinfectant against a range of microbial agents. Here we investigate the ability of Virusend to inactivate SARS-CoV-2. Using surface and solution inactivation assays, we show that Virusend is able to reduce SARS-CoV-2 viral titre by 4 log_10_ p.f.u. ml^−1^ within 1 min of contact. Ensuring disinfectants are highly effective against SARS-CoV-2 is important in eliminating environmental sources of the virus to control the COVID-19 pandemic.

## Introduction

Severe acute respiratory syndrome coronavirus 2 (SARS-CoV-2) is a novel coronavirus that is the causative agent of COVID-19 which first emerged in late 2019 [1]. Countries are working to control transmission of SARS-CoV-2 with the ultimate goal of production and large-scale manufacture of effective vaccines [2–4]. Until an effective vaccine is widely distributed, control of the virus is limited to implementing measures such as contact tracing, quarantine, enforcing strict social distancing, advising frequent hand hygiene and infection control measures in hospital environments [5]. During the 2002 outbreak of SARS-CoV-1, and the 2012 Middle East respiratory syndrome-related (MERS)-CoV outbreak, virus stability on environmental surfaces facilitated transmission events [6]. Similarly, research has shown that SARS-CoV-2 can remain viable on surfaces, notably plastic and stainless steel for up to 72 h post-inoculation, and in aerosols for at least 3 h, meaning effective disinfectants can prevent indirect contact transmission [7]. Virusend (TX-10) has been developed to work as a highly effective disinfectant that rapidly inactivates enveloped viruses. As communities begin to reopen and people return to the workplace, effective and quick disinfection of communal areas is paramount to maintaining control of COVID-19. Here we present the evidence that Virusend can reduce SAR-CoV-2 virus within 1 min both in solution and on surfaces.

## Methods

### Cell culture and viruses

Vero E6 cells (C1008: African green monkey kidney cells), obtained from Public Health England, were maintained in Dulbecco’s minimal essential medium (DMEM) containing 10 % foetal bovine serum (FBS) and 0.05 mg ml^−1^ gentamicin. Cells were kept at 37 °C with 5 % CO_2_. Passage four or five of a SARS-CoV-2 isolate (REMRQ0001/Human/2020/Liverpool) from a clinical sample was used to assess inactivation of Virusend. On the fourth and fifth passages the virus was cultured in Vero E6 cells maintained in DMEM with 4 % FBS and 0.05 mg ml^−1^ gentamicin at 37 °C and 5 % CO_2_ as previously described [8]. The fifth passage of the virus was harvested 48 h after inoculation and concentrated by passage through a centrifugal column (Amicon Ultra-15 100 kDa MWCO). Virus was used immediately after concentrating.

### Virus inactivation

Inactivation on surfaces were performed using either 9.8 log_10_ or 7.9 log_10_ p.f.u. ml^−1^ of SARS-CoV-2. Surface inactivation was carried out by inoculating the hard surface, stainless steel discs, with 50 µl of virus and allowed to air dry at room temperature for 1 h. Dried inoculum was incubated with 100 µl of Virusend (TX-10; Virusend was developed by Pritchard Spray Technologies, Colchester, UK) or autoclaved water for control samples for either 30 s or 9.5 min, after which 900 µl of DMEM containing 2 % FBS and 0.05 mg ml^−1^ gentamicin was added and mixed until dried inoculum was dissolved. The sample was then transferred into a dilution series for virus quantification at exactly 1 min or 10 min after addition of Virusend to the dried inoculum. Solution inactivation assays used either 8.4 log_10_ or 7.9 log_10_ p.f.u. ml^−1^ and were carried out by incubating 25 µl of inoculum with 100 µl of Virusend or autoclaved water for control samples for either 1 min or 10 min. After incubation, 10 ml of DMEM was added and transferred to a dilution series within 30 s of DMEM being added. All experiments were performed in duplicate.

### Cytotoxicity assay

Cytotoxicity for surface inactivation was determined by inoculating stainless steel discs with 50 µl of DMEM containing 2 % FBS and 0.05 mg ml^−1^ gentamicin and allowed to air dry at room temperature for 1 h. Dried inoculum was incubated with 100 µl of Virusend or autoclaved water for 5 min, after which 900 µl of DMEM containing 2 % FBS and 0.05 mg ml^−1^ gentamicin was added and mixed until dried inoculum was dissolved. The sample was then transferred into a dilution series and a standard plaque assay performed. Cytotoxicity for solution assays were performed by incubating 25 µl of DMEM containing 2 % FBS and 0.05 mg ml^−1^ gentamicin with 100 µl of Virusend for 5 min, after which 10 ml of DMEM was added and sample transferred to a dilution series for standard plaque assays. The cytotoxicity assays were performed in duplicate.

### Suppression assay

Suppression for solution inactivation was assayed by adding 25 µl of inoculum, either 8.4 log_10_ or 7.9 log_10_ p.f.u. ml^−1^, to 100 µl of Virusend in 10 ml of DMEM and incubated for 30 s. After 30 s, the sample was transferred into a dilution series and a standard plaque assay performed. The suppression assay was performed in duplicate.

### Virus quantification and viability

Samples from each condition were serial diluted 10-fold for quantification by standard plaque assay using Vero E6 cells [9]. Cells were incubated for 72 h at 37 °C and 5 % CO_2_, then fixed with 10 % formalin and stained with 0.05 % crystal violet solution. Plaques were counted to calculate virus titre. All samples were performed in technical duplicates.

## Results

**Fig. 1.**
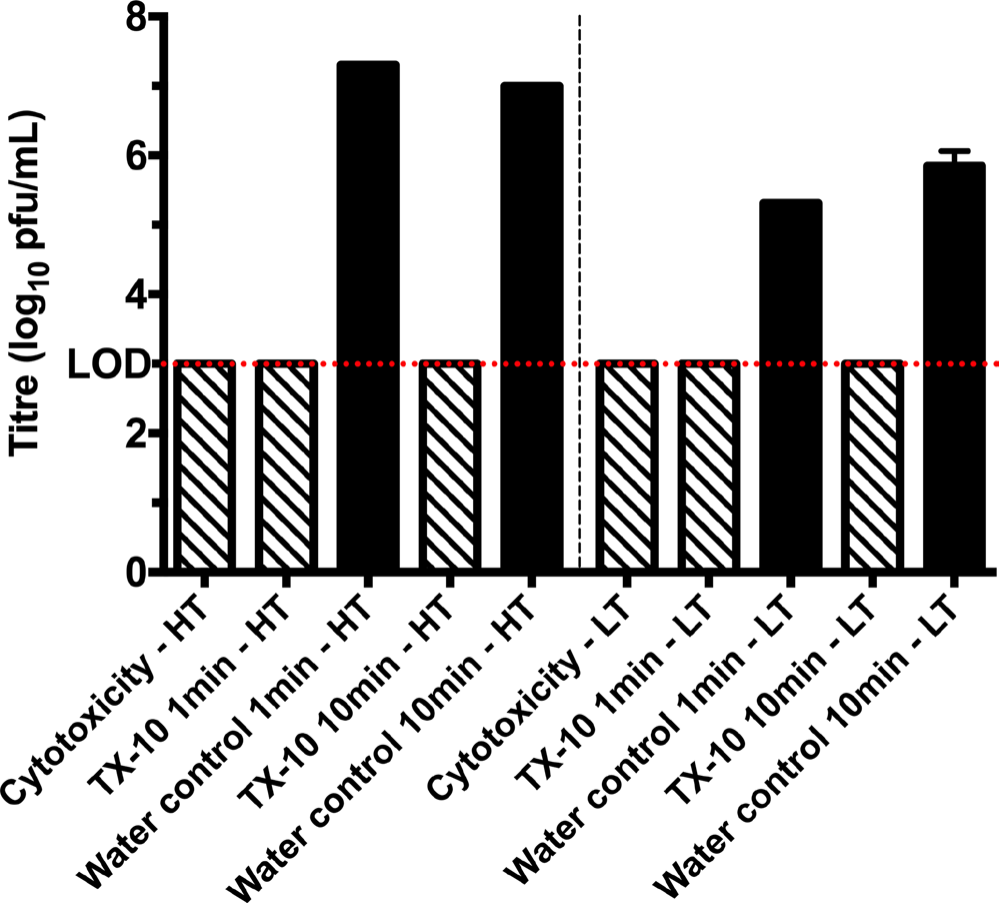
Virusend (TX-10) reduces viral titre on hard surfaces by at least 4.0 log_10_ p.f.u. ml^−1^ with high titre (HT) viral inoculum after contact times of 1 min and 10 min. When low titre (LT) inoculum was used, Virusend reduces virus titre by at least a 2.3 log_10_ p.f.u. ml^−1^ at both 1 min and 10 min contact time. Diagonal pattern represents cytopathic effect caused by TX-10 and solid black represents the titre of infectious virus following each treatment. Limit of detection (LOD) (3.0 log_10_ p.f.u. ml^−1^) is shown across the graph with a dotted red line. All columns represent mean of *n*=2,±SD.

For inactivation assays, Virusend was directly placed on SARS-CoV-2 inoculum, for an incubation period of either 1 min or 10 min. On the hard surface (stainless steel disc), contact time of 1 min with Virusend reduced SARS-CoV-2 titres to below the limit of detection for both high and low titre inoculum (Fig. 1). A titre of 7.3 log_10_ p.f.u. ml^−1^ was recovered from the high titre, hard surface control samples. Similarly, incubation with Virusend for 10 min reduced the virus titre to below the limit of detection, compared with 7.0 log_10_ p.f.u. ml^−1^ recovered from the high titre control. With a low titre inoculum, Virusend also reduced SARS-CoV-2 titres to below the limit of detection after contact times of 1 and 10 min on hard surfaces. Titres of 5.3 log_10_ p.f.u. ml^−1^ and 5.9 log_10_ p.f.u. ml^−1^ were recovered from the 1 and 10 min control samples, respectively. Cytotoxicity assays with Virusend in the absence of virus were used to determine the limit of detection, the point at which Vero E6 cell death is due to the cytotoxicity of Virusend, and not virus. Cytopathic effect was observed to 3.0 log_10_ p.f.u. ml^−1^ (Fig. 1). Both inactivation and cytotoxicity assays confirm a reduction of at least 4.0 log_10_ p.f.u. ml^−1^ of infectious SARS-CoV-2 with high titre inoculum and a reduction of at least 2.3 log_10_ p.f.u. ml^−1^ with low titre inoculum (Fig. 1).

**Fig. 2.**
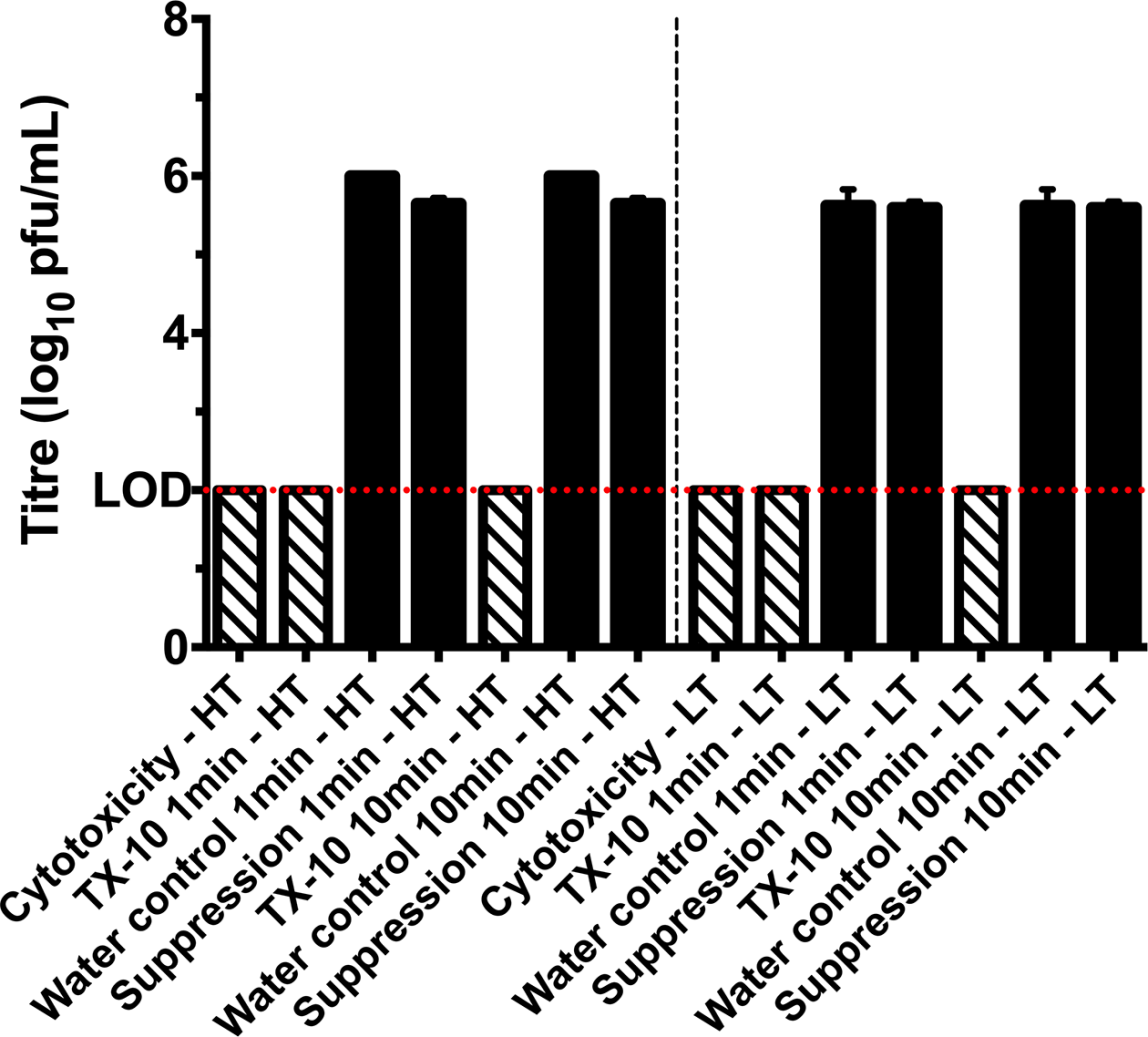
Virusend (TX-10) reduces viral titre in solution by at least 4.0 log_10_ p.f.u. ml^−1^ when incubated with high titre (HT) virus inoculum for 1 min and 10 min. When low titre (LT) inoculum was used, both incubation periods reduced the titre by at least 3.6 log_10_ p.f.u. ml^−1^, to below the limit of detection. Diagonal pattern represents cytopathic effect caused by Virusend and solid black represents the titre of infectious virus following each treatment. Limit of detection (LOD) (2.0 log_10_ p.f.u. ml^−1^) is shown across the graph with a dotted red line. All columns represent mean of *n*=2,±SD.

For inactivation assays in solution, Virusend was placed directly into solution with SARS-CoV-2 for either 1 or 10 min. An incubation period of 1 min with Virusend reduced the high titre inoculum by 4.0 log_10_ p.f.u. ml^−1^, from 6.0 log_10_ p.f.u. ml^−1^ in the water control to below the limit of detection (Fig. 2). A 10 min incubation with Virusend also reduced viral titre by 4.0 log_10_ p.f.u. ml^−1^, from 6.0 log_10_ p.f.u. ml^−1^ to below the limit of detection. With the low titre inoculum, the addition of Virusend reduced SARS-CoV-2 to below the limit of detection at both 1 min and 10 min incubation times, reductions of 3.6 log_10_ p.f.u. ml^−1^ (Fig. 2). Titres of 5.6 log_10_ p.f.u. ml^−1^ were recovered from control samples at 1 min and 10 min. A suppression assay for solution inactivation assays was used to demonstrate that dilution with 10 ml of DMEM suppressed Virusend inactivation of SARS-CoV-2 upon the completion of the assay. The addition of virus inoculum to Virusend in 10 ml of DMEM recovered a virus titre of 5.7 log_10_ p.f.u. ml^−1^ with high titre inoculum and 5.6 log_10_ p.f.u. ml^−1^ with low titre inoculum. Cytotoxicity assays for solution inactivation assays showed the limit of detection for these assays was 2.0 log_10_ p.f.u. ml^−1^.

## Discussion

SARS-CoV-2 can remain viable on surfaces, notably plastic and stainless steel, for up to 72 h post-inoculation, and in aerosols for at least 3 h [7]. In solutions, SARS-CoV-2 may remain viable for up to 14 days at 4 °C, 7 days at room temperature, and for 1 to 2 days at 37 °C [10]. Therefore, contaminated surfaces and solutions are a reservoir for transmission through fomites, meaning effective hygiene and environmental decontamination is crucial in helping to prevent the spread of COVID-19 [11, 12]. Disinfectant solutions of 75 % ethanol and 10 % sodium hypochlorite are able to reduce SARS-CoV-2 titre by at least 2.0 log_10_ TCID_50_ ml^−1^ and 3.25 log_10_ TCID_50_ ml^−1^, respectively, within 5 min [10, 13]. However, the World Health Organization (WHO) has recommended diluting household bleach 1 : 100 to reduce irritation to the user and contact times of 10 to 60 min to disinfect surfaces and when immersing items [14]. Rapid household disinfectants could reduce transmission in private residence and public spaces, such as offices. Detergents, such as NP-40 and Triton X-100, have been shown to inactivate SARS-CoV-2 at a concentration of 0.5 % [8] and inactivate the enveloped hepatitis C virus to below detectable levels at even lower concentrations within 1 min [15]. However, environmental concerns over Triton X-100 use have resulted in calls to produce alternative products (https://echa.europa.eu/authorisation-list). Virusend is also a detergent-based disinfectant, containing N-(3-Aminopropyl)-N-dodecylpropane-1,3-diamine. Here we have shown that Virusend is able to reduce SARS-CoV-2 virus titre by at least 4.0 log_10_ p.f.u. ml^−1^ in 1 min of contact time making it an effective disinfectant for households and public spaces.

An initial obstruction to the work presented here, was the need for a high virus titre to show a 4.0 log_10_ p.f.u. ml^−1^ reduction due to the cytotoxicity of Virusend to Vero E6 cells. The limit of detection indicated the point at which cytopathic effect in Vero E6 cells is caused by Virusend and not the virus. Therefore, to achieve a 4.0 log_10_ p.f.u. ml^−1^ reduction, the SARS-CoV-2 culture supernatant had to be concentrated after harvesting to give stock titres of 8.4 log_10_ and 9.8 log_10_ p.f.u. ml^−1^. When a lower stock virus titre of 7.9 log_10_ p.f.u. ml^−1^ was used, a 4.0 log_10_ p.f.u. ml^−1^ reduction could not be demonstrated and would not meet the strict requirements of European Standard testing. However, these assays still showed a similar trend of inactivation. The reduction with high titre virus stock indicates the effectiveness of Virusend, which may be necessary to inactivate SARS-CoV-2 in environments that are contaminated [16].

Disinfectants tested for use against other members of *Coronaviridae* have typically used surrogates to carry out the assays more easily. One example of a surrogate virus is murine hepatitis virus, a lower risk group pathogen that can be grown to high titres and has structural and genetic similarities to SARS-CoV [17]. Surrogates are chosen to mimic the target virus during inactivation, but the use of surrogates should be limited, and the target pathogen should be used when possible [18]. Here we have been able to test Virusend against an isolate of SARS-CoV-2 collected from a human infection and assess the ability of Virusend to significantly reduce the titre of the relevant virus.

Current advice focuses on increasing public engagement to encourage essential control measures, such as maintaining high levels of hygiene in the home [19]. Virusend can reduce the strain of demand on current hygiene product resources, to be used within private residences, communal public areas such as offices and hospital environments [20–22]. The efficacy against SARS-CoV-2 during a short contact time make it suitable for rapid disinfection of contaminated surfaces and solutions. The development of disinfectants such as Virusend and others is important as we continue efforts to reduce transmission of SARS-CoV-2.
